# Exploring the Differences in the Gut Microbiome in Atopic Dermatitis According to the Presence of Gastrointestinal Symptoms

**DOI:** 10.3390/jcm11133690

**Published:** 2022-06-27

**Authors:** Chang-Yi Han, Soon-Kyeong Kwon, Mijung Yeom, Dae-Hyun Hahm, Jae-Woo Park, Hi-Joon Park, Kyuseok Kim

**Affiliations:** 1 Department of Ophthalmology, Otorhinolaryngology, and Dermatology of Korean Medicine, Graduate School of Korean Medicine, Kyung Hee University, Seoul 02447, Korea; dolphin072680@gmail.com; 2 Division of Applied Life Science (BK21), Gyeongsang National University, Jinju 52828, Korea; skkwon@gnu.ac.kr; 3 Acupuncture & Meridian Science Research Center, College of Korean Medicine, Kyung Hee University, Seoul 02447, Korea; myeom@khu.ac.kr (M.Y.); dhhahm@khu.ac.kr (D.-H.H.); 4 Department of Physiology, School of Medicine, Kyung Hee University, Seoul 02447, Korea; 5 Department of Gastroenterology, College of Korean Medicine, Kyung Hee University, Seoul 02447, Korea; pjw2907@khu.ac.kr; 6 Department of Ophthalmology, Otorhinolaryngology, and Dermatology of Korean Medicine, College of Korean Medicine, Kyung Hee University, Seoul 02447, Korea

**Keywords:** atopic dermatitis, gastrointestinal symptoms, gut microbiome, gut–skin axis

## Abstract

(1) Background: Atopic dermatitis (AD) is a multifactorial chronic allergic skin disease. Gastrointestinal (GI) functions have been suggested to be associated with its incidence or severity. As modulators of the gut–skin axis, gut microbes might affect the pathophysiology of AD. (2) Methods: We divided a cohort of patients with AD according to their GI symptoms as follows: AD with epigastric fullness (ADwEF), AD with epigastric rigidity (ADwER), and AD without GI symptoms (ADw/oGI). The gut microbial profiles were analyzed using 16S rRNA amplicon sequencing. (3) Results: The microbiota of the ADwER group showed low diversity indices in richness and evenness and formed a separate cluster to the other groups. In the ADwER group, the proportion of *Bacteroides* increased, while that of *Prevotella* decreased; functional pathways related to phosphotransferase systems were not abundant relative to those in the ADw/oGI group. Taken together, patients with AD with GI symptoms have a different microbiome from patients with simple AD. (4) Conclusions: In an exploratory study aimed at evaluating the relationship between AD and GI symptoms, the gut microbiome in patients with AD with GI symptoms differed from that in patients with simple AD, and this result could serve as a basis for further gut–skin axis studies.

## 1. Introduction

Atopic dermatitis (AD) is a common chronic allergic inflammatory disease with a prevalence of up to 15–30% in infants and 10% in adults [1]. Genetic, environmental, and immunological factors, as well as skin barrier dysfunction, are known to be involved in the pathophysiology of AD.

As the gut microbiome has emerged as the major regulator of the gut–skin axis, recent studies on the gut microbiota and patients with inflammatory skin diseases, including AD, have been conducted [1,2,3,4,5]. The gut microbiota is a large collection of microorganisms in the human gastrointestinal (GI) tract. It contributes to the host’s health in various aspects, including contribution to the integrity of the GI epithelium barrier by producing short-chain fatty acids (SCFAs) and mediation of immune response by peptidoglycan regulating the Toll-like receptors [6,7]. The importance of gut microbiota diversity has been highlighted in recent studies examining the association between the gut microbiota and chronic inflammatory skin diseases, such as acne, rosacea, and psoriasis, as well as AD [3,4]. A recent systematic literature review that analyzed the association of inflammatory skin diseases with imbalance of the gut microbiota found that there were consistently significant differences in the gut microbiota between healthy individuals and patients with acne vulgaris, psoriasis, and chronic urticaria [5]. However, it was reported that studies on AD have shown conflicting results [5].

Several studies have reported that the proportions of *Clostridia*, *Clostridium difficile*, *Escherichia coli*, and *Staphylococcus aureus* in the guts of patients with AD are higher than those in the guts of healthy individuals; meanwhile, the proportions of *Lactobacillus* and *Bifidobacterium* and overall microbial diversity are reduced [8,9]. According to a clinical study on the improvement of SCORing Atopic Dermatitis (SCORAD) indexes in children with AD through a probiotic *Lactobacilli* analysis [10], *Lactobacillus* preparations are currently used as therapeutic aids for AD [6]. Another study reported an increase in the proportions of *Faecalibacterium prausnitzii* subspecies, which might be associated with reductions in the proportions of SCFAs, such as propionate, acetate, and butyrate, which damage GI epithelial barrier integrity and induce abnormal Th2 immune response [7,11]. These studies have revealed the association of the gut microbiota with AD; however, they could not show the differences in gut microbiota according to the clinical GI features in patients with AD.

In the British Health Improvement Network study [12], the prevalence of AD was significantly higher in patients with functional dyspepsia (FD), irritable bowel syndrome (IBS), and functional gastrointestinal disorder (FGID). As GI symptoms and skin symptoms pathophysiologically share a common pathology of Th2 immune response in each GI mucosa and derma [2,13], they are likely to accompany one another at a high rate. However, to date, few studies have clarified the association between AD and GI symptoms.

In our previous clinical studies [14,15], we have found that the SCORAD index indicated a significantly more severe condition in patients with AD with epigastric rigidity (ADwER) than in those with AD without GI symptoms (ADw/oGI) [14]; further, the SCORAD index improved, and gastric discomfort was alleviated through acupuncture treatment [15].

Based on these findings, we hypothesized that GI symptoms would be associated with AD symptoms and that differences would be associated with differences in the gut microbiota. Therefore, the aim of this study was to explore the differences in gut microbiota structures, including composition, richness, evenness, distribution, distinguishing taxa, and pathways, between patients with AD with and without GI symptoms.

## 2. Materials and Methods

### 2.1. Eligibility Criteria

The inclusion/exclusion criteria were as follows:

*Inclusion criteria (Healthy group)*

- Men and women 19 to 49 years old;
- 18.5 < BMI ≤ 25;
- No AD according to the Hanifin and Rajka criteria [16];
- Agreement with the study protocol and willingness to sign written informed consent.

*Inclusion criteria (AD group)*

- Men and women 19 to 49 years old;
- 18.5 < BMI ≤ 25;
- AD diagnosed according to the Hanifin and Rajka criteria;
- Scores from 30 to 80 points on a 100 mm Visual Analog Scale (VAS) for pruritus (0, no symptoms at all; 100, worst possible symptoms).
- Agreement with the study protocol and willingness to sign written informed consent.

*Exclusion criteria (Healthy group and AD group)*

- Treatments administered that may affect results e.g., (antibiotics, non-steroidal anti-inflammatory drugs (NSAIDs), PPIs, antidepressants) for 3 months before collection of the fecal sample;
- Systemic disease e.g., (hypertension, diabetes mellitus, liver disease, thyroid disease, arrhythmia, hemorrhagic disease, stroke, rheumatoid arthritis);
- Consumption of dairy products, including probiotics, or meat and fried foods in more than one meal per day within 7 days before collection of the fecal sample;
- Smoking;
- Drinking within the last week;
- Physical and mental problems that the Korean medical doctor (KMD) considers inappropriate;
- Participation in other clinical studies that may affect this study.

### 2.2. Subject Recruitment

A total of 30 subjects (20 patients with AD and 10 healthy individuals as a reference) were selected from patients who visited Kyung Hee Medical Center. The sample size was determined via an expert consensus.

All subjects were divided into four groups according to the presence of AD and GI symptoms identified through abdominal examination: ADwER, AD with epigastric fullness (ADwEF), ADw/oGI, and no AD and GI symptoms (HSw/oGI). In the holistic view of traditional Korean medicine, patients with AD tend to complain of epigastric fullness and rigidity. The GI symptoms were evaluated by the same investigator.

In Korean medicine, abdominal examination supposes the status of GI organs via touching and pressing of the abdomen and evaluation of signs, such as mass, pain, rigidity, and distention [17,18]. Epigastric rigidity was confirmed via palpation, while epigastric fullness was confirmed on the basis of the subjects’ feeling of being always full regardless of food consumption [14,19]. Epigastric fullness reflects GI muscle swelling due to temporary tissue fluid retention; meanwhile, epigastric rigidity, which is the worsened state of epigastric fullness, reflects GI hardening due to chronic dysfunction and impaired nutrition supply [14,19].

Before fecal sampling, a questionnaire was administered to confirm the usual eating habits of the subjects. The questionnaire was modified by referring to a questionnaire previously used in the literature (Appendix A) [20,21]. 

### 2.3. 16S rRNA Gene Sequencing and Analysis

Fecal samples were collected using disposable, sterile, and spoilage-preventing fecal kits (WHPM, Irwindale, CA, USA) and stored at room temperature (20 ± 5 °C) at the Korean Medicine Clinical Trial Center. Total genomic DNA was extracted from 2000~3000 mg of fecal sample aliquot using a Herculase II Fusion DNA Polymerase Nextera XT Index Kit V2, following the manufacturer’s instructions. PCR targeting the V3–V4 region of the 16S rRNA gene was conducted with forward (5′-TCGTCGGCAGCGTCAGATGTGTATAAGAGACAGCCTACGGGNGGCWGCAG-3′) and reverse (5′-GTCTCGTGGGCTCGGAGATGTGTATAAGAGACAGGACTACHVGGGTATCTAATCC-3′) primer sets; the amplicons were sequenced via the Illumina MiSeq instrument using 2 × 250 bp paired-end reads. Microbial profiling was conducted mainly using the Qiime2 version 2021.2, with the DADA2 package as a denoising package. The EzTaxon database was used as a taxonomic reference database. Any sequences classified as members of Archaea were removed to focus primarily on the bacterial community. Generated taxonomy tables, phylogenetic trees, and metadata were imported into the R environment or Agile Toolkit for Incisive Microbial Analyses (http://atima.jplab.net/, accessed on 1 May 2022) for further analysis or visualization. Functional abundances based on the 16S rRNA gene sequencing data were inferred using the PICRUSt2 software [22].

### 2.4. Statistical Analysis

The counted number of observed operational taxonomic units (OTUs) and Shannon and inverse Simpson diversity indices were selected as the indicators of alpha diversity. Weighted UniFrac phylogenetic distance matrices were used to compute beta diversity. Furthermore, the permutational multivariate analysis of variance (PERMANOVA) test was performed using the adonis function in the Vegan R package with 999 permutations. Differential taxonomic and functional abundances were assessed using the linear discriminant analysis effect size (LEfSe) method [23]; the logarithmic LDA threshold score was set at 2.0. The Mann–Whitney U test was applied when comparing variables of two categories; the Kruskal–Wallis test was applied when comparing multiple groups. Statistical analysis was performed using R Statistical Software, version 3.6.1 (R Foundation for Statistical Computing, Vienna, Austria).

## 3. Results

### 3.1. Cohort Characteristics among the ADw/oGI, ADwEF, ADwER, and HSw/oGI Groups

A total of 30 participants (20 patients with AD and 10 healthy subjects) were included. However, 3 out of the 10 healthy fecal samples were excluded because the quality control failed during DNA library production. Therefore, 27 fecal samples were analyzed.

According to the classification criteria, the subjects were divided as follows: ADw/oGI (*n* = 7), ADwEF (*n* = 7), ADwER (*n* = 6), and HSw/oGI (*n* = 7). The general characteristics, demographic information, questionnaire findings, objective SCORAD indexes [24], and VAS scores (pruritus) are shown in Table 1. Differences were found in age and family history of allergic disorders, but not in other characteristics.

### 3.2. Profiling of the Gut Microbiota According to AD and Accompanying GI Symptoms

**Figure 1:**
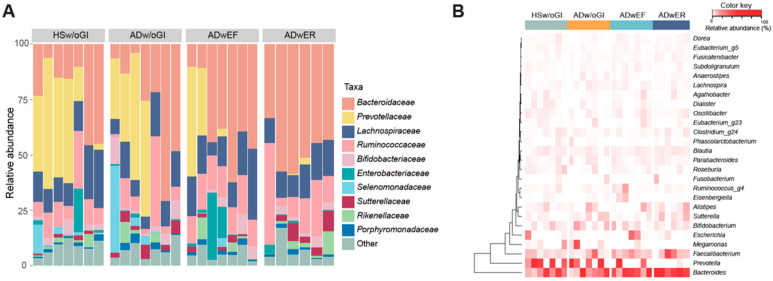
Compositional features of the gut microbiota in the healthy individuals and in the patients with atopic dermatitis (AD). (**A**) Taxonomic profile of the subjects at the family level. (**B**) Heatmap of the relative abundances of the most abundant genera. HSw/oGI, healthy subjects without gastrointestinal symptoms; ADw/oGI, patients with AD without gastrointestinal symptoms; ADwEF, patients with AD with epigastric fullness; ADwER, patients with AD with epigastric rigidity.

We assessed the microbial community composition and structure by targeting the 16S rRNA V3–V4 regions via amplicon sequencing. The sequences were predominantly assigned to the *Bacteroidaceae*, *Prevotellaceae*, *Lachnospiraceae*, *Ruminococcaceae*, *Bifidobacteriaceae*, and *Enterobacteriaceae* families. The *Bacteroidaceae* family was the most abundant family in patients with AD and the second most abundant family, followed by the *Prevotellaceae* family, in the healthy subjects (Figure 1A).

At the genus level, *Bacteroides* belonging to the *Bacteroidaceae* family, *Prevotella* (*Prevotellaceae* family), *Faecalibacterium* (*Ruminococcaceae* family), *Bifidobacterium* (*Bifidobacteriaceae* family), and *Escherichia* (*Enterobacteriaceae* family) were the most successfully detected genera in the fecal samples. In particular, the genera *Prevotella* and *Megamonas* were mostly detected in the ADw/oGI and HSw/oGI groups; meanwhile, the genera *Bacteroides*, *Bifidobacterium*, and *Faecalibacterium* were mostly detected in the ADwEF and ADwER groups (Figure 1B).

**Figure 2:**
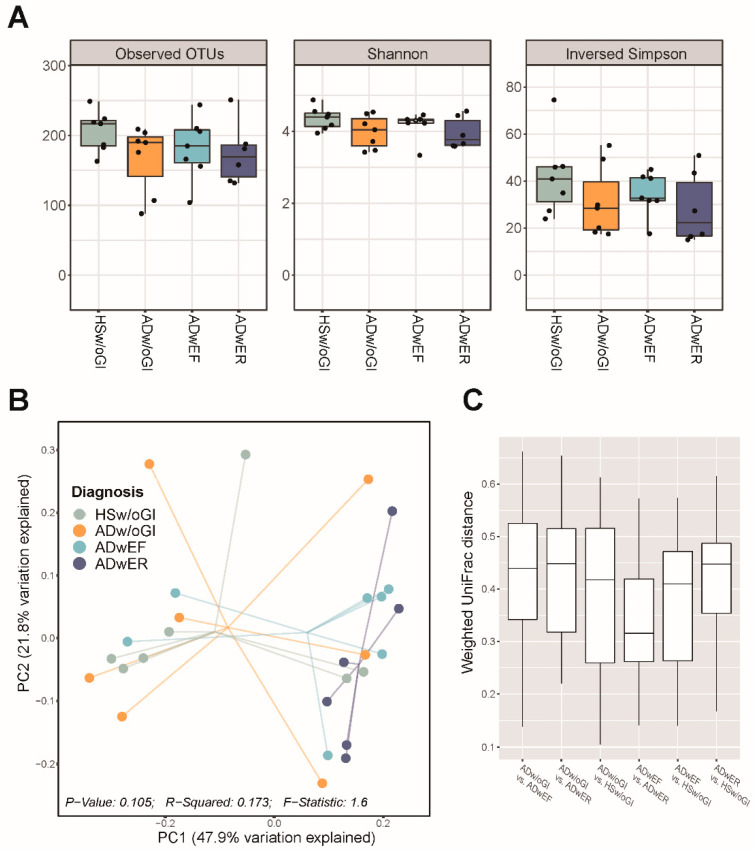
Diversity of the gut microbiota in the healthy individuals and in the patients with atopic dermatitis (AD). (**A**) Alpha diversity in the healthy individuals and patients with AD based on the number of observed operational taxonomic units (OTUs) and Shannon and inverse Simpson indices. (**B**) Principal coordinate analysis (PCoA) of the weighted UniFrac distances of the gut microbial communities represented by the diagnosis and (**C**) the UniFrac distances between the groups.

Next, we evaluated alpha diversity, which was measured using the Shannon and inverse Simpson indices and observed OTUs. Observed OTU richness proportionally decreased as AD or GI symptoms worsened; however, there were no significant differences between the groups. Similarly, the Shannon and inverse Simpson evenness indices tended to be lower in the ADwER group and higher in the HSw/oGI group than in the other groups (Figure 2A).

A principal coordinate analysis (PCoA) plot based on the UniFrac distance matrix revealed that the ADwER group had a relatively clustered distribution; the ADwEF group had a distribution close to that of the ADwER group, except for two samples (Figure 2B), and the weighted UniFrac distances between the microbial compositions were closest in the ADwEF and ADwER groups (Figure 2C). The pairwise PERMANOVA test conducted between the groups also revealed that the ADwER group showed findings significantly different from those in the HSw/oGI (*p* = 0.014) and ADw/oGI groups (*p* = 0.037).

### 3.3. Microbiota Comparison According to GI Symptoms in the Patients with AD

**Figure 3:**
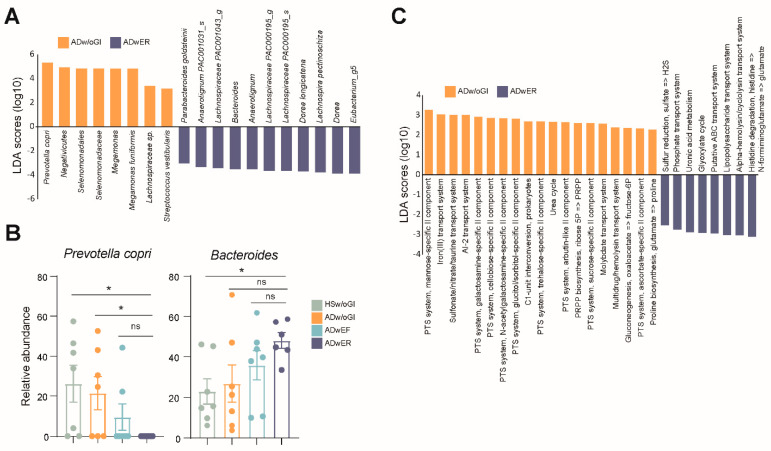
Microbiota comparison according to the gastrointestinal (GI) symptoms in the patients with atopic dermatitis (AD). (**A**) Microbial taxa associated with the AD diagnoses. (**B**) Relative abundance of *Prevotella copri* and genus *Bacteroides*. The differences in the means were tested using the Mann–Whitney U test. * *p* < 0.05; ns, non-significant. (**C**) Enriched metabolic sets in terms of KEGG modules in the microbiota of the patients with AD with different GI symptoms.

To further evaluate the microbiota according to GI symptoms in the patients with AD, we compared the microbiota between the ADwER and ADw/oGI groups. We retrieved the taxa that contributed most to the differences between the groups in the LEfSe analysis. In this analysis, 19 taxa that were relevant to each group were identified (Figure 3A). In the ADw/oGI group, the enriched species were *Prevotella copri*, *Megamonas funiformis*, and *Streptococcus vestibularis*; however, the relative abundance of *Stereptococcus vestibularis* was <1% in both groups. Meanwhile, *Prevotella copri* (22.74%) and *Megamonas funiformis* (4.16%) were some of the dominant taxa in the ADw/oGI group, and no sequence reads matching these two species were detected in the ADwER group. Other enriched taxa in the ADwER group were the genera *Eubacterium_g5*, *Dorea*, *Anaerotignum*, *Lachnospira pectinoschiza*, *Dorea longicatena*, and *Parabacteroides goldsteinii*; however, their relative abundances were all <1%.

In particular, the relative abundance of *Prevotella copri* decreased proportionally as AD and GI symptoms worsened (Figure 3B). In the ADwER group, the most meaningful taxon among the enriched taxa was *Bacteroides*, which was the major taxon constituting the gut microbiota (48.5% in the ADwER group and 31% in the ADw/oGI group); its proportion also increased as AD and GI symptoms worsened.

Next, we evaluated the microbial functional pathway in terms of KEGG modules enriched in the ADwER and ADw/oGI groups using the PICRUSt2 software pipeline (Figure 3C). Interestingly, the KEGG modules involved in the transport of metabolites were significantly different between the two groups. Particularly in the ADw/oGI group, many functional pathways related to phosphotransferase (PTS) systems were enriched. Transport systems, including iron (III), sulfonate/nitrate/taurine, AI-2, molybdate, and multidrug/hemolysin systems, were more abundant in the ADw/oGI group than in the ADwER group. Meanwhile, transport systems, including alpha-hemolysin/cyclolysin, lipopolysaccharide, ABC transport, and phosphate systems, were more enriched in the ADwER group than in the ADw/oGI group.

## 4. Discussion

Gastrointestinal symptoms, such as abdominal distension, the feeling of incomplete evacuation, and straining, occurred more frequently in patients with AD than in the healthy controls [25]. It was also reported that the prevalence of AD was significantly higher in patients with FD, IBS, and FGID [15]. Recent studies have reported that, as modulators of the gut–skin axis, GI microorganisms can influence the pathophysiology of AD [1,26]. Further, an unbalanced increase in the rate of allergic diseases, including AD, may be related to the typical Western diet, i.e., a low-fiber, high-fat diet [2]. The Western diet results in a deficiency of SCFAs, which alters the genes of GI microbes and causes an inflammatory response in the GI tract and skin [2]. In our previous clinical studies [14,15], we have found that the severity of AD was higher in AD patients with epigastric rigidity than in those with AD without GI symptoms. However, there was no study on whether there was a difference in the gut microbiota according to the presence or severity of digestive symptoms. In this study, we evaluated the relationship between AD and GI symptoms and found that the gut microbiome in patients with AD with GI symptoms differs from that in patients with simple AD.

Considering the evidence of GI microorganisms as modulators of the gut–skin axis, we attempted to explore the composition and functional pathway of the gut microbiome according to the presence or absence of GI symptoms (epigastric fullness and epigastric rigidity) in patients with AD.

In our study, the gut microbiota taxonomy abundance ratio was different between the groups; particularly in the ADwER group, the proportion of *Prevotella* was remarkably low, while the proportion of *Bacteroides* was relatively high. The analysis of alpha diversity showed no difference in each group; however, the patients in the ADwER and ADwEF groups had lower richness and evenness indices than the patients with ADw/oGI. In particular, the number of observed OTUs and Shannon and inverse Simpson indices were the lowest in the ADwER group, indicating decreased microbial diversity and severe imbalance therein. The analysis of beta diversity showed some differences in each group. The PCoA plots showed a scattered distribution in each group; however, the ADwER group showed a relatively clustered linear pattern; the ADwEF group showed a distribution close to that of the ADwER group; and the weighted UniFrac distances between the microbial compositions were closest in the ADwEF and ADwER groups.

The enriched functional pathways between the ADwER and ADw/oGI groups were different. The PTS pathway was enriched in the ADw/oGI group, which was different to what was found in the ADwER group, reflecting the results for gut microbiota. The PTS system controls various physiological processes but has two main functions. First, it mediates ATP production through transport and phosphorylation of carbohydrates and regulates cellular processes; it also guarantees an optimal supply of energy without taking up too many carbon sources [27,28]. Second, the PTS system regulates the virulence of certain pathogens because some virulence genes affect carbon catabolite repression [27]. An association between PTS system activity and GI metabolism and the dominant taxa *Prevotella* has not been reported; however, in one case reporting changes in GI microbial composition and metabolic functions after bariatric surgery [29], PTS system activity increased, and it was presumed that this compensated for the decreased nutrient uptake after the bypass.

The distinguishing taxa between the ADwER and ADw/oGI groups were *Prevotella copri* (dominant in the ADw/oGI group) and *Bacteroides* (dominant in the ADwER group) in the LEfSe analysis. This was consistent with the taxonomy abundance ratio. In addition, they were inversely related; when the proportion of *Prevotella* decreased, that of *Bacteroides* increased, as AD and GI symptoms worsened. *Bacteroides* and *Prevotella* both belong to the *Bacteroidetes phylum* and are believed to have a common ancestor [30]; however, they are antagonistic to each other [29] and distinct in terms of enterotypes [31]. *Prevotella* and *Bacteroides* also have distinct dietary characteristics. *Prevotella* represents a major cluster in a fruit- and vegetable-rich diet, while *Bacteroides* represents a major cluster in a protein- and animal fat-rich diet [31]. In one study [32], children from rural African villages had a higher proportion of *Prevotella* than children from Italian cities and it was assumed that African children have adapted to extract the maximum amount of energy from vegetable and fiber-rich environments; thus, *Prevotella*, breaking down fiber and producing by-product SCFAs, is dominant. In another study comparing the prevalence of colon cancer in 12 African Americans and 12 native Africans [33], Americans who consumed a high-protein diet had a high proportion of *Bacteroides*, while native Africans who consumed a high-complex carbohydrate diet had a high proportion of *Prevotella*. In a Cochrane review of AD, dietary supplements, e.g., fish oil, vitamin D, and vitamin E, were evaluated as effective, though this remains questionable [34]. Nevertheless, diet is a factor that is relatively easier to control when managing patients with AD compared with other environmental and genetic factors. In a cross-sectional study examining patient reports and perceptions after diet control in patients with AD [35], 47.6% of AD symptoms improved when vegetables were added to the diet; this was attributed to the abundant supply of carotenoids and flavonoids, which are inversely associated with various oxidative stress parameters and inflammatory cytokines. Therefore, additional studies are needed to understand the interactions between diet, gut microflora, and skin.

This study had several limitations. As an exploratory study, this study divided a small number of 27 subjects into four groups. A normal distribution was not obtained; thus, it was difficult to confirm the significance of the results. An appropriate number of groups and subjects should be selected in future studies, considering the possibility of fecal sample drop-out and statistical significance. Additionally, if allergy tests (IgE level or eosinophil count) were performed; additional GI symptom questionnaires and more specific diet-related questionnaires were administered; and associations with microbial indicators were analyzed, more meaningful results could have been obtained.

Despite these limitations, this study is meaningful in that, from a gut microbiome perspective, it provides evidence of the association between AD and GI symptoms. The composition of microorganisms in the patients with AD with GI symptoms was different from that in the patients with simple AD, which might be attributed to the distinguishing taxa of *Prevotella* and *Bacteroides*, the dominant genera in the GI tract. Therefore, additional research is needed to reveal the direct causality and relevant detail that would allow the aforementioned limitations to be overcome.

## Figures and Tables

**Table 1:** Cohort information.

	HSw/oGI	ADw/oGI	ADwEF	ADwER	
	(*n* = 7)	(*n* = 7)	(*n* = 7)	(*n* = 6)	*p*-Value
Age (y)	29.1 ± 4.5	22.0 ± 0.6	24.6 ± 3.7	22.2 ± 2.0	0.001
Sex					0.09
Female	2 (28.6%)	5 (71.4%)	2 (28.6%)	5 (83.3%)	
Male	5 (71.4%)	2 (28.6%)	5 (71.4%)	1 (16.7%)	
Height (cm)	174.0 ± 9.6	163.9 ± 8.4	173.7 ± 6.7	164.6 ± 9.4	0.058
Weight (kg)	68.1 ± 10.3	61.0 ± 8.9	67.8 ± 9.1	60.4 ± 9.5	0.301
Body mass index (kg·m^−2^)	22.4 ± 1.6	22.6 ± 1.5	22.3 ± 1.5	22.3 ± 3.0	0.993
Bristol stool scale					0.415
Constipation (type 1–2)	0 (0.0%)	2 (28.6%)	1 (14.3%)	0 (0.0%)	
Normal (type 3–5)	7 (100.0%)	5 (71.4%)	5 (71.4%)	5 (83.3%)	
Diarrhea (type 6–7)	0 (0.0%)	0 (0.0%)	1 (14.3%)	1 (16.7%)	
Objective SCORAD index	(-)	27.0 ± 9.4	22.2 ± 4.9	30.4 ± 8.6	0.19
VAS score (pruritus)	(-)	5.9 ± 0.9	5.5 ± 1.0	5.8 ± 1.5	0.80

HSw/oGI, healthy subjects without gastrointestinal symptoms; ADw/oGI, patients with AD without gastrointestinal symptoms; ADwEF, patients with AD with epigastric fullness; ADwER, patients with AD with epigastric rigidity; SCORAD, SCORing Atopic Dermatitis. Continuous data were analyzed by the Kruskal–Wallis test and presented as means ± standard deviation. Binary data were analyzed by the chi-square test and presented as numbers of percentiles (%).

## Data Availability

The data that support the findings of this study are available from the corresponding author upon reasonable request.

## Appendix A. Questionnaire about Diet, Sleep, Family History of Allergic Disease, Social History, and History of Other Systemic Diseases

**(1) Diet**		
Did you eat breakfast for more than 3 days in the last week?	Yes □	No □
Did you drink milk for more than 3 days in the last week?	Yes □	No □
Did you eat cheese for more than 3 days in the last week?	Yes □	No □
Did you eat bread for more than 3 days in the last week?	Yes □	No □
Did you eat cake for more than 3 days in the last week?	Yes □	No □
Did you eat vegetable for more than 3 days in the last week?	Yes □	No □
Did you eat meat for more than 3 days in the last week?	Yes □	No □
Did you eat ham for more than 3 days in the last week?	Yes □	No □
Did you eat fish for more than 3 days in the last week?	Yes □	No □
**(2) Sleep**		
Did you sleep for more than 6 h in the last week?	Yes □	No □
Did you sleep before 24 o’clock in the last week?	Yes □	No □
**(3) Family history of allergic disease**		
Do you have a family member with allergic rhinitis, asthma, or atopic dermatitis?	Yes □	No □
**(4) Social history**		
Do you smoke?	Yes □	No □
Did you drink in the last week?	Yes □	No □
**(5) History of other systemic diseases**		
Do you have any diseases, such as hypertension, liver disease, cardiac disease, cerebral infarction, or rheumatoid arthritis?	Yes □	No □
Do you have any endocrine diseases, such as diabetes mellitus or thyroid disease?	Yes □	No □
